# Comparison of Open Microscopic and Biportal Endoscopic Approaches in Multi-Level Posterior Cervical Foraminotomy: Radiological and Clinical Outcomes

**DOI:** 10.3390/jcm14010164

**Published:** 2024-12-30

**Authors:** Hyung Rae Lee, Jae Min Park, In-Hee Kim, Jun-Hyun Kim, Jae-Hyuk Yang

**Affiliations:** 1 Department of Orthopedic Surgery, Korea University Anam Hospital, Seoul 02841, Republic of Korea; drhrleeos@gmail.com (H.R.L.); jk497@cornell.edu (J.-H.K.); 2 College of Medicine, Korea University, Seoul 02841, Republic of Korea; kikara02@korea.ac.kr; 3 National Police Hospital, Seoul 05715, Republic of Korea; soaringss@naver.com

**Keywords:** posterior cervical foraminotomy (PCF), open microscopic, biportal endoscopic spine surgery (BESS), surgical route

## Abstract

**Background/Objectives:** This study compares clinical and radiological outcomes of open microscopic posterior cervical foraminotomy (PCF) and biportal endoscopic spine surgery (BESS) PCF in multi-level cases. While BESS PCF is effective in single-level surgeries, its role in multi-level procedures remains unclear. **Methods**: This retrospective cohort study included 60 patients treated for cervical radiculopathy from 2016 to 2023, divided into two groups, open microscopic PCF (Group M, *n* = 30) and BESS PCF (Group B, *n* = 30). Clinical outcomes were assessed using visual analogue scale (VAS) scores for neck and arm pain and the neck disability index (NDI). Radiological parameters included cervical angle, segmental angle, range of motion (ROM), and the extent of facetectomy. **Results**: Both groups showed improvement in the arm pain VAS and the NDI. However, Group B exhibited significantly better neck pain on the VAS at the final follow-up (*p* = 0.03). Radiologically, Group B maintained lordotic cervical and segmental angles postoperatively, while Group M showed kyphotic changes (*p* < 0.01). Segmental ROM was larger in Group M, indicating greater instability (*p* < 0.01). Group B had less extensive facetectomy while achieving comparable foraminal enlargement. Operative time was longer for Group B (*p* < 0.001). **Conclusions**: BESS PCF preserves cervical stability and reduces postoperative neck pain compared to open microscopic PCF in multi-level procedures. Despite longer operative times, its benefits in minimizing instability make it a promising option for treating multi-level cervical radiculopathy. Further research with long-term follow-up is recommended.

## 1. Introduction

Cervical radiculopathy, caused by nerve root compression, involves symptoms such as pain, tingling sensations, and muscle weakness localized to the region supplied by the affected nerve root [1,2,3]. When conservative managements are not effective, surgical treatments can be considered like other degenerative spinal disorders [4,5]. Posterior cervical foraminotomy (PCF) has been acknowledged be an effective treatment [6,7]. PCF achieves root decompression by surgical approaches including resection of the superior articular process, herniated disc, and hypertrophied ligaments [2].

PCF can be performed either through an open microscopic surgery or by biportal endoscopic spine surgery (BESS). PCF using BESS has advanced quickly and demonstrated clinical and radiological outcomes comparable to open microscopic PCF [8]. Particularly in single-level radiculopathy, BESS PCF has been reported that it has advantages over open microscopic surgery in relation to shorter operating times, reduced blood loss, and decreased postoperative length of stay [9,10,11]. On the other hand, when the level of surgery increases to more than two, the time and effort required for minimally invasive surgery increases proportionally. This is because BESS PCF, unlike open microscopic surgery, does not dissect multiple levels at once but requires a new approach for each additional level. In this reason, surgeons tend to prefer open microscopic surgery over BESS in multi-level PCF. However, open microscopic PCF includes significant muscle stripping and retraction, which cause significant postoperative pain, impaired muscle function, and cervical dynamic instability [12,13,14].

Unfortunately, while comparative studies exist for single-level procedures, there is a lack of research comparing clinical or radiological outcomes related to changes in cranial dimensions and postoperative cervical instability between open microscopic PCF and BESS PCF in multi-level procedures. Therefore, in this study, we aimed to compare open microscopic surgery and BESS in multi-level PCF regarding clinical and radiographic outcomes.

## 2. Materials and Methods

### 2.1. Patients

This retrospective cohort study was approved by the institutional review board (IRB no. 2024AN0349 and approval date 8 May 2024) and adhered to the ethical guidelines of the World Medical Association Declaration of Helsinki. Obtaining informed consent was waived due to the retrospective study design. This study complied with the guidelines of the Strengthening the Reporting of Observational Studies in Epidemiology (STROBE) for cohort studies [15,16]. In total, 212 patients who underwent open microscopic PCF or BESS PCF as the treatment for cervical radiculopathy between August 2016 and June 2023 were investigated for eligibility. PCF was selected in cases where radiculopathy was the primary symptom, and the pathology was limited to foraminal stenosis or laterally located soft disc protrusions without significant ventral compression. Patients with unilateral radiculopathy who expressed concerns about anterior approaches due to neck scarring or implant-related discomfort were also considered for PCF. Cases with significant cervical instability or central stenosis, such as myelopathy caused by ossification of the posterior longitudinal ligament, were excluded. The decision between open microscopic PCF and BESS-PCF was made through thorough discussions with the patient. Factors considered during these consultations included the patient’s preference regarding surgical scars, the potential risks associated with each approach, and the anticipated operative time. Patients were provided with comprehensive explanations of both surgical techniques to ensure an informed decision. All surgeries were performed by a single spine surgeon (H.R.L.) with expertise in cervical spine procedures. Exclusion criteria were as follows: (1) patients who underwent single-level surgery or combined anterior and posterior surgery; (2) patients with infection, trauma, tumours, or preoperative instability; and (3) patients with a history of previous cervical spinal surgery.

**Figure 1:**
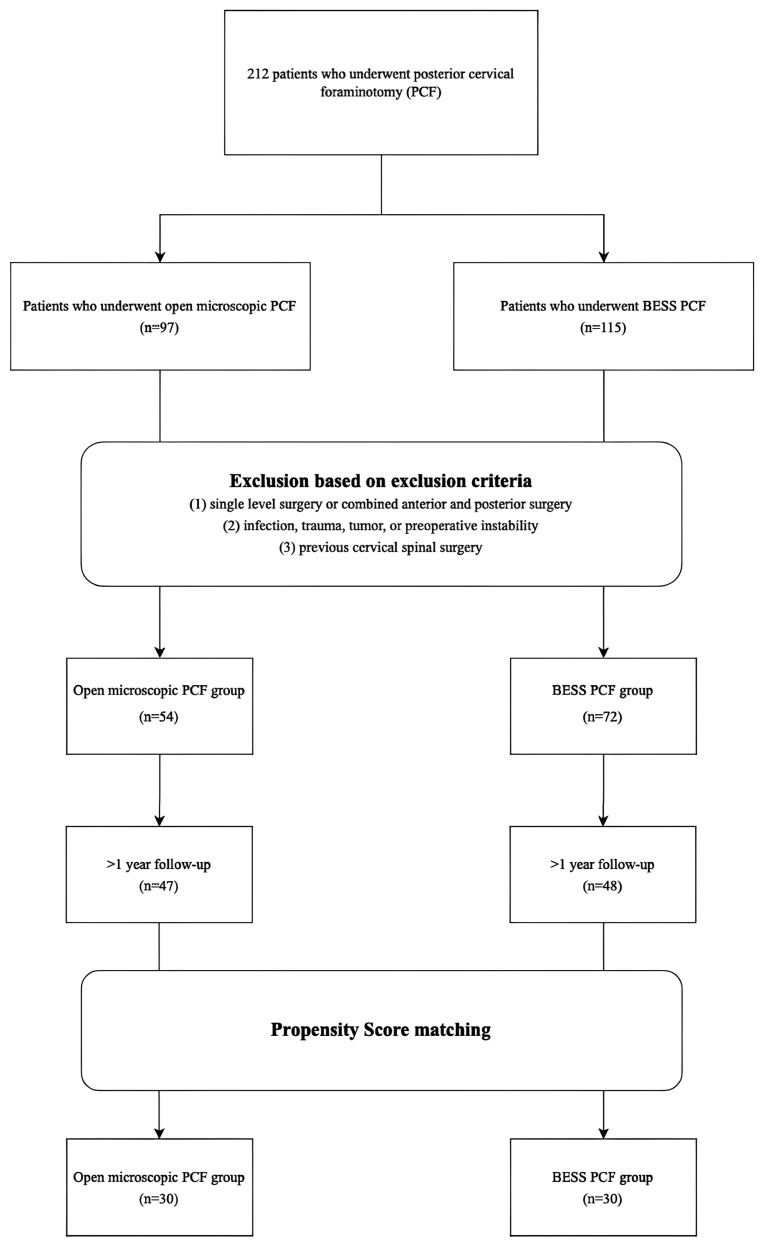
Patient selection process.

To ensure a more reliable comparison between the groups, propensity score matching was performed based on age, sex, and BMD (bone mineral density), resulting in a final cohort of 30:30. Consequently, our study included 60 patients who received posterior cervical foraminotomy (PCF) at our hospital for unilateral radiculopathy caused by degenerative cervical disease. The open microscopic PCF group (M) consisted of patients who underwent open microscopic PCF for cervical radiculopathy, and the BESS PCF group (B) consisted of those who underwent BESS PCF. We reviewed their medical records, with all patients having undergone at least a 1-year follow-up that included clinical and radiological assessments. Demographic and radiological information was collected from electronic medical records and the picture-archiving communication system (PetaVision for Clinics, 3.1, Korea University Anam Hospital, Seoul, Korea) (Figure 1).

### 2.2. Surgical Procedure

#### 2.2.1. Open Microscopic PCF

Open microscopic PCF was performed under general anesthesia with the patient’s head in a prone position, slightly flexed and stabilized using Mayfield clamps. A longitudinal skin incision of appropriate length for the targeted level was made on the unilateral side, and the midline posterior ligament complex was preserved. After confirming the appropriate surgical level with fluoroscopic guidance, resection of the inferior articular process of the upper vertebra and the superior articular process of the lower vertebra was performed using high-speed drills. Unlike laminectomy, which can compromise posterior spinal structures and increase the risk of iatrogenic instability or kyphosis, PCF preserves critical elements such as the spinous process, posterior longitudinal ligament, and a substantial portion of the facet joint. In our study, we ensured that more than 50% of the facet joint was preserved during unilateral foraminotomy, as this has been shown to maintain cervical stability even in multi-level cases. Furthermore, our cohort excluded patients with pre-existing instability or significant risk factors for postoperative kyphosis, further reducing the likelihood of instability-related complications. The exiting nerve root was visualized and decompressed. In cases where extruded or ruptured disc material was present, it was carefully removed using pituitary forceps. The neural foramen was assessed for adequate decompression with a Freer elevator. For additional levels, the same technique was applied. After completing the procedure, the wound was closed, and a suction drain was placed, typically removed the next day.

#### 2.2.2. BESS PCF

BESS PCF procedures were conducted using a standard biportal endoscopic system equipped with a 4.0 mm diameter zero- or 30-degree optic endoscope. Precision surgical instruments, including 1.0 mm and 2.0 mm Kerrison punches and a 1.5 mm pituitary forceps, were utilized. Two portals were established over the adjacent pedicles of the target level with the aid of serial dilators and a working sheath. The initial target was the medial border of the facet joint, where soft tissues were cleared with a radiofrequency probe. Laminotomy and foraminotomy were performed using a diamond drill, confirming the nerve root. The trajectory was adjusted according to the location of the lesion, and the position of the portals was fine-tuned to align with this direction. This allowed for optimal visualization and access to the target area. To minimize facet resection, the lateral foraminal decompression was executed through this mediolaterally oriented surgical approach. Like open microscopic procedures, for additional lesion levels, we utilized the portal from the adjacent segment and created one additional portal for each additional level to perform multi-level PCF.

### 2.3. Outcome Assessment

Patient-reported outcomes, including the neck disability index (NDI, out of 50) and visual analogue scale (VAS) scores for the neck (NP, out of 10) and arm (AP, out of 10) were documented preoperatively, as well as at 2 months and 1 year following surgery.

### 2.4. Radiological Measurement

All preoperative plain radiographs were taken with the patients in neutral, flexion, and extension neck positions. For the neutral view, patients stood with a horizontal gaze, while for the flexion and extension views, they were asked to flex or extend their neck to a comfortable limit [17,18]. Radiographic follow-up was conducted at 2 and 12 months using the same protocol. The radiographic parameters assessed included the segmental Cobb angle at the surgical level (SA), the C2−C7 Cobb angle (CA), T1 slope, C2−C7 sagittal vertical axis (SVA), range of motion of C2−C7 (CROM), range of motion at the operative level (SROM), SA in flexion (SAF), SA in extension (SAE), CA in flexion (CAF), and CA in extension (CAE). Lordotic angles were recorded as negative and kyphotic angles as positive.

**Figure 2:**
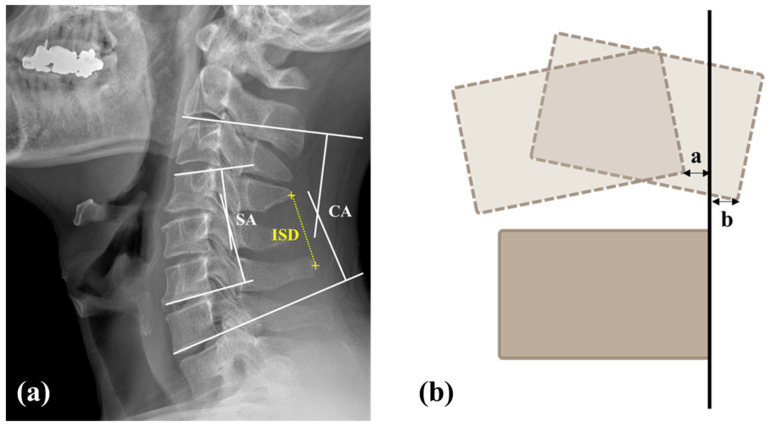
Radiologic measurements. (**a**) The segmental angle (SA) was the Cobb’s angle between the inferior endplate of the lower vertebrae and superior endplate of the upper vertebrae. The cervical angle (CA) was the angle between lines which were parallel to the trailing edge of C2 and C7. The interspinous distance (ISD) was defined as the distance between midpoints of edges of the spinous processes. (**b**) The gliding distance of the cervical spine (GD) at the corresponding disc level in flexion and extension positions. Gliding distance of cervical spine = a (flexion) + b (extension).

**Figure 3:**
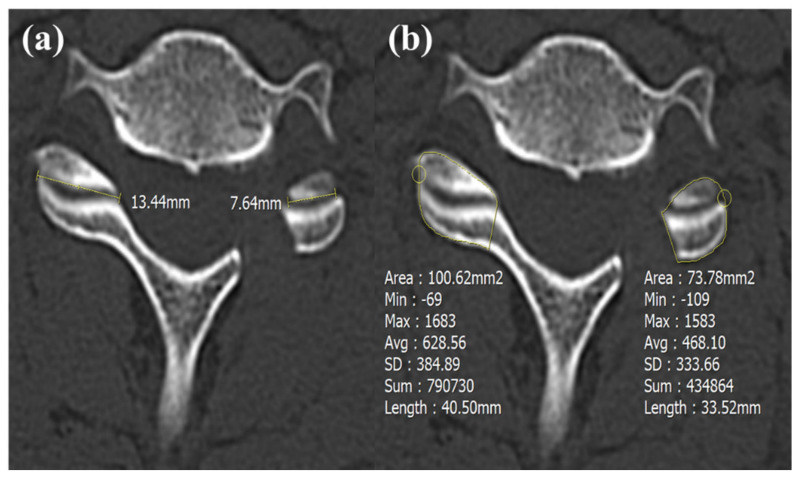
Methods for measuring the extent of facetectomy. The extent of facetectomy was evaluated using two methods, namely (**a**) comparing the length of the contralateral facet with the remaining facet length after facetectomy and (**b**) comparing the area of the contralateral facet with the remaining facet area after facetectomy.

Cervical disc degeneration was assessed using the Pfirrmann classification, which grades disc degeneration on T2-weighted preoperative cervical MRI images into five categories [19,20]. On a flexion–extension lateral dynamic radiograph, gliding distances were measured in both flexion and extension positions (Figure 2) [21]. To investigate the facetectomy amount, the area and length of the contralateral facet (A) and the residual facet (B) were measured on postoperative CT scans. The facetectomy percentage was calculated with the following formula: Facetectomy (%) = (A − B) × 100/A (Figure 3) [22]. The extent of joint resection was evaluated in the axial plane, reconstructed parallel to the disc level in accordance with our standard CT protocol.

Radiological measurements were independently conducted by two examiners (J.M.P. and H.R.L.) who were blinded to the patients’ clinical information and were not involved in their treatment. The reliability of these measurements was evaluated using the intraclass correlation coefficient (ICC). The ICC values for interobserver reliability were 0.85, 0.87, 0.83, and 0.91 for C2–C7 SVA, cervical angle during flexion and extension, facetectomy percentage, and Pfirrmann grade, respectively.

### 2.5. Statistical Analysis

A comparison of radiological and clinical outcomes between Groups M (open microscopic PCF) and B (BESS PCF) was performed. The independent *t*-test and chi-squared test were applied to analyze continuous and categorical variables, respectively. Time-dependent data were analyzed by repeated-measures (RM) ANOVA, followed by post hoc comparisons between the two groups. Bonferroni adjustments, including all pairwise comparisons within a specific model, were applied to *p*-values for multiple testing. Post hoc comparisons between the main effects of all pairs of time points were performed. All statistical analyses were performed using IBM SPSS Statistics version 21.0 for Windows (IBM Corp., Armonk, NY, USA). Statistical significance was set at *p <* 0.05.

## 3. Results

### 3.1. Clinical Outcomes

This study involved a total of 60 patients, with 30 patients each in Group B (male, *n* = 21; female, *n* = 9) and Group M (male, *n* = 23; female, *n* = 7). The demographic data including age, sex ratio, bone mineral density (BMD), diabetes mellitus (DM), smoking, and coronary artery disease (CAD) were not significantly different between Group B and Group M (Table 1). The two-level surgery was the most in both groups (Group B, *n* = 19 (63.3%); Group M, *n* = 16 (53.3%)) and the four-level was only in Group M (*n* = 2 (6.7%)). The operated spinal levels were C4–5 (*n* = 9), C5–6 (*n* = 24), C6–7 (*n* = 24), and C7–T1 (*n* = 14) in Group B and C4–5 (*n* = 12), C5–6 (*n* = 26), C6–7 (*n* = 24), and C7–T1 (*n* = 14) in Group M. There were no significant differences in pathological type, 24H-drainage, and hospital day between Group B and Group M. However, the mean operation time in Group M was significantly lower than in Group B (Group B, 84.3 ± 11.9; Group M, 61.7 ± 13.2; *p* < 0.001) (Table 1).

**Figure 4:**
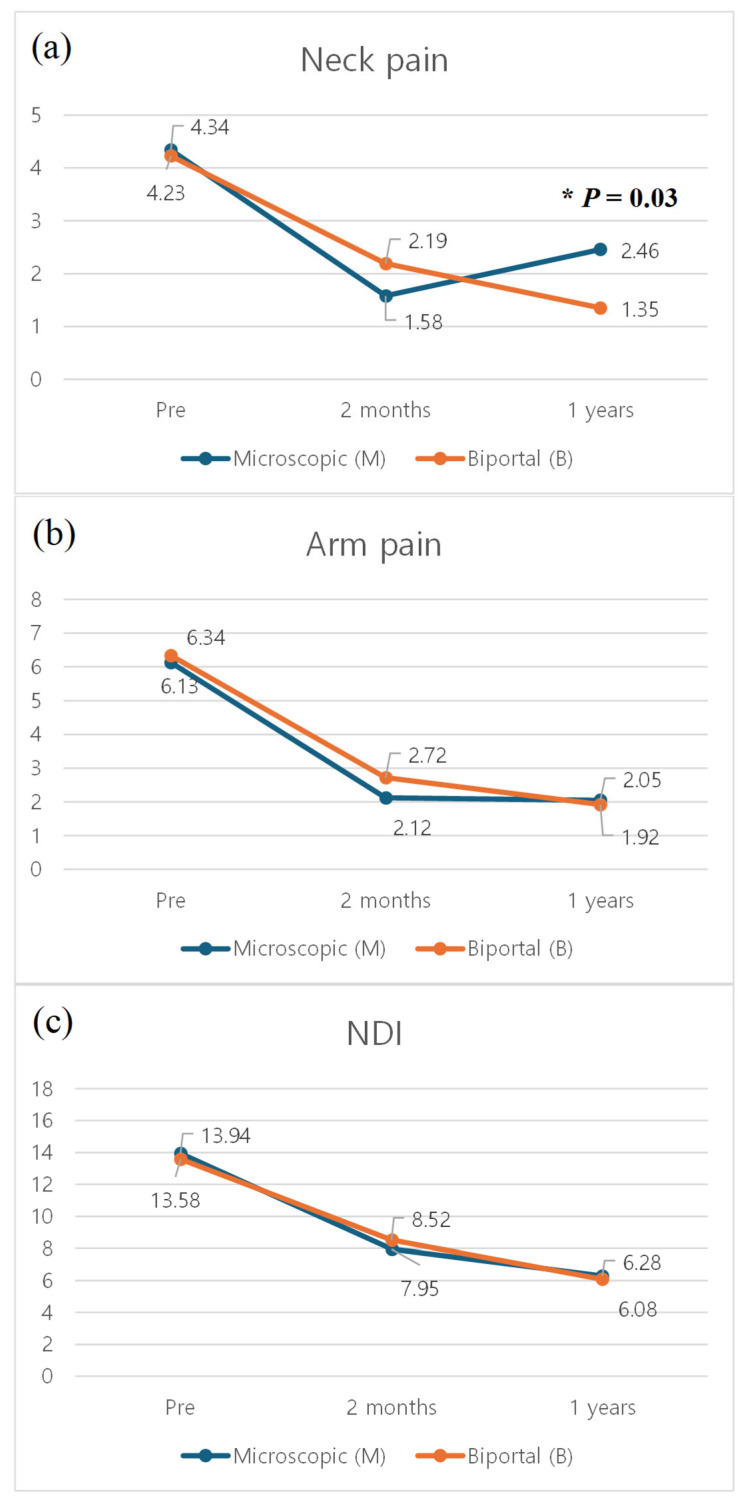
Comparisons of the mean (**a**) neck pain VAS, (**b**) arm pain VAS, and (**c**) NDI over time between Groups E and O. VAS, visual analogue scale; NDI, neck disability index. * *p*-value < 0.05.

Regarding clinical outcomes, both groups demonstrated improvement following surgery. There was no difference between the two groups in terms of the arm pain VAS and the NDI at every time point. In the neck pain VAS, Group M showed more adverse outcomes than Group B at final follow-up (Figure 4).

### 3.2. Radiological Assessment

**Figure 5:**
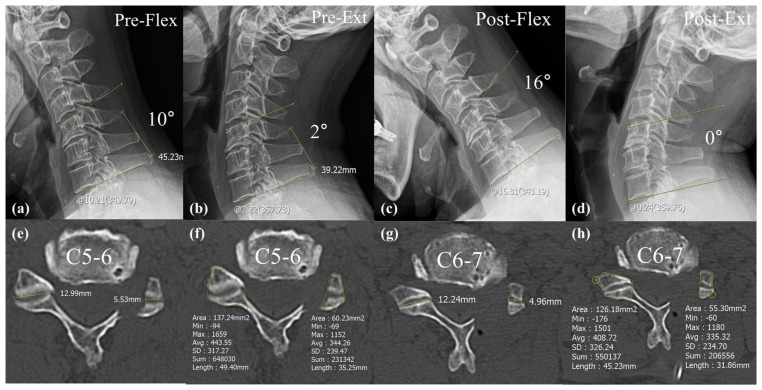
Representative patient from Group O. (**a**,**b**) Preoperative flexion cervical lateral radiograph showing a 10-degree kyphotic angle at C5–C7 and an extension lateral radiograph showing a 2-degree kyphotic angle. Therefore, the preoperative segmental ROM is 8 degrees. (**c**,**d**) Postoperative flexion cervical lateral radiograph showing 16 kyphotic degrees at C5–C7 and an extension lateral radiograph showing 0 degrees. Therefore, the postoperative segmental ROM is 16 degrees, resulting in a segmental ROM gain of 8 degrees. (**e**,**f**) The amount of facetectomy at C5–6 was measured by length (57.4%) and area (56.1%). (**g**,**h**) The amount of facetectomy at C6–7 was measured by length (59.4%) and area (56.1%).

Preoperatively, there were no significant differences in segmental angle, cervical angle, C2–7 SVA, CROM, and SROM between two groups. In both the segmental angle and cervical angle, Group B showed more lordotic angle than Group M postoperatively at the 2 months (segmental, *p* = 0.02; cervical, *p* < 0.01) and final follow-up (segmental, *p* = 0.01; cervical, *p* < 0.01) (Table 2). The cervical angle decreased from −9.0 ± 10.1 preoperatively to −11.8 ± 11.5 and −13.0 ± 12.3 at 2 months and 1 year postoperatively, respectively, in Group B. Conversely, in Group M, the cervical angle increased from −5.7 ± 9.1 preoperatively to −4.7 ± 8.8 at the last follow-up. Group M showed significantly larger segmental ROM than Group B postoperatively at 2 months (*p* = 0.01) and 1 year (*p* < 0.01) (Table 2). An example of increased segmental ROM after surgery is illustrated in Figure 5. However, a significant difference in cervical ROM was not observed among the two groups postoperatively.

**Figure 6:**
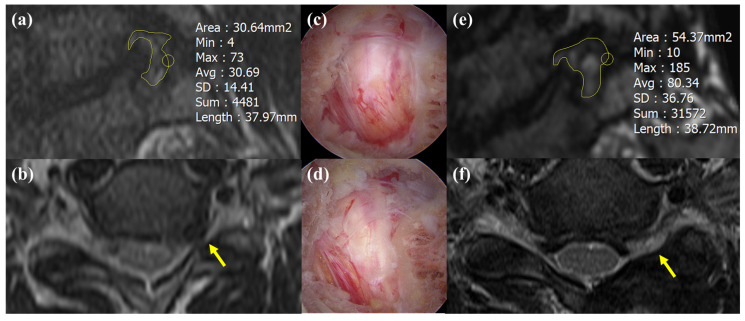
Preoperative and postoperative imaging and surgical process for biportal endoscopic spine surgery (BESS) in multi-level posterior cervical foraminotomy. (**a**,**b**) Preoperative magnetic resonance imaging (MRI) of the cervical spine, showing the foraminal and axial views. The foraminal area was measured using PACS software, calculated as 30.64 mm^2^. The yellow arrow indicates the left foramen. (**c**,**d**) Intraoperative views during a two-level cervical foraminotomy using the BESS system, illustrating the precise decompression process. (**e**,**f**) Postoperative MRI demonstrating the increased foraminal area (54.47 mm^2^) in the foraminal view and the widened foramen in the axial view, confirming successful decompression. The yellow arrow indicates the left foramen.

**Figure 7:**
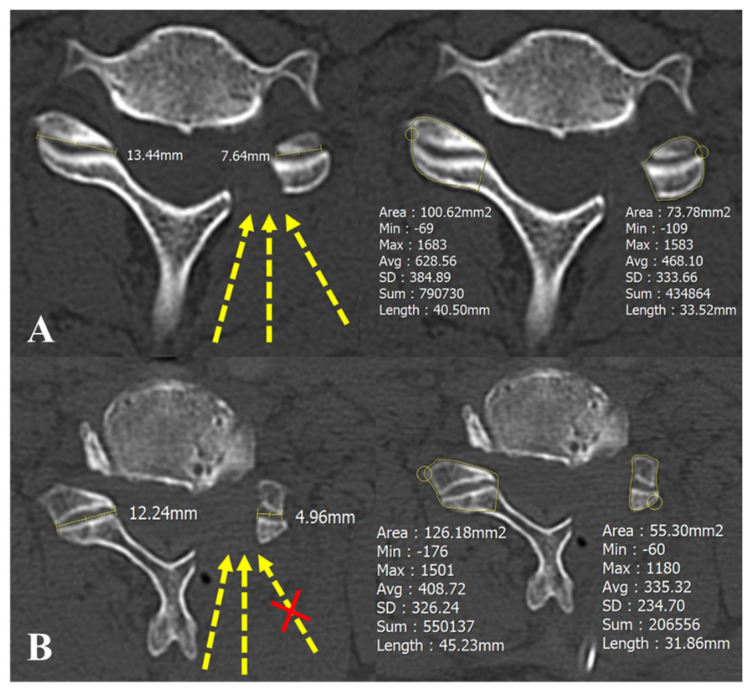
The available surgical routes were drawn to the facet joint on the postoperative CT images. (**A**,**B**) A representative patient from Group E and O, respectively. (**A**) The lateromedial surgical route was available in BESS PCF. (**B**) The lateromedial surgical route was restricted in open microscopic PCF.

The disc height, gliding distance, and Pfirrmann grade were not significantly different between two groups. The means and standard deviations of the preoperative and postoperative foraminal dimension and enlargement are presented in Table 3. In Group B, the foraminal dimension was 31.5 ± 7.8 (mm^2^) preoperatively and 53.3 ± 7.4 (mm^2^) postoperatively. The foraminal enlargement was 75.5 ± 34.6 (%) in Group B. In Group M, the foraminal dimension was 34.3 ± 8.5 (mm^2^) preoperatively and 54.5 ± 8.2 (mm^2^) postoperatively. The foraminal enlargement was 63.8 ± 26.1 (%) in Group M. There was no statistical difference in terms of foraminal enlargement. The ratio of bone bridge formation among Group M was significantly higher than among Group B (*p* = 0.02). In Group B, the facetectomy amount, measured by area and length, was less than in Group M (length, *p* < 0.01; wide, *p* < 0.01) (Table 3) (Figure 6). Interestingly, while there was no difference in foraminal enlargement between the groups, Group B had preserved more facet that Group M. Figure 7 shows a lateromedial surgical route that was restricted in Group M.

### 3.3. Complications

The comparison of complications between Group B and Group M revealed no significant differences in the incidence of complications between the two groups (Table 4). Specifically, the occurrence of incomplete decompression was one case in Group B and two cases in Group M (*p* = 0.57), while hematoma occurred in one case in each group. Other complications, such as wound infection, local recurrence, postoperative seizures, and delirium, were either rare or absent in both groups. Overall, the total number of complications was three in Group B and four in Group M, with no statistically significant difference (*p* = 0.68).

## 4. Discussion

The goal of the present study was to compare open microscopic PCF and BESS PCF in multi-level procedures. Our results represent an important understanding of cervical curvature change after each PCF and focus on cervical instability by facet resection.

Several studies have shown that there were no differences in neck pain VAS scores between open microscopic PCF and BESS PCF after 2 months postoperatively [9,23,24]. For example, a study Fessler et al. said that there was no difference in neck pain improvement between open foraminotomy and endoscopic foraminotomy [25]. However, our findings are contrary to them. At the last follow-up, the VAS scores for the neck pain of patients who underwent BESS PCF were lower than that of patients who underwent open microscopic PCF (Figure 5). In multi-level procedures, it is likely to improve neck pain more significantly than open microscopic PCF for cervical radiculopathy. Several reasons can be inferred for this. Firstly, as shown in Figure 7, open microscopic PCF results in more severe facet violation compared to BESS PCF due to limitations in the surgical route. Excessive facet joint destruction during PCF may lead to concerns about post-decompression segmental instability, which can result in neck pain [26,27]. Secondly, open microscopic PCF requires extensive dissection of the entire posterior musculature, which is likely to result in significant postoperative pain [28,29]. Moreover, these effects are likely to be even more pronounced in multi-level cases as opposed to single-level procedures.

Open posterior approaches detach the laminae and spinous processes’ extensor cervical muscles, which can lead to loss of lordosis or even spinal instability [14,30,31]. While open surgery allows for easier spatial navigation by exposing surrounding anatomical landmarks, it is not without its limitations. Endoscopic surgery, although magnified, uses similar instruments to open surgery. With the aid of saline mixed with epinephrine, nerve roots can often be identified more easily, particularly their distal portion, axilla, and superior and inferior boundaries. Preoperative MRI allows for planning the extent of decompression, which is then guided intraoperatively by anatomical landmarks such as the facet joint, V-point, and lateral end of the facet. These features, coupled with the ability to adjust the surgical route (Figure 7), ensure that incomplete decompression is not an inherent limitation of endoscopic methods.

However, multi-level procedures, especially at three levels, require repeating the muscle preparation process, increasing fatigue and necessitating advanced surgical skills. Despite this, our findings indicate no significant differences in decompression or foraminal enlargement between the groups. From an alignment perspective, the differences observed likely result from factors such as muscle stripping and facet violation, which are more pronounced in open surgery. These findings underscore the importance of considering both the surgical technique and postoperative outcomes when selecting an approach. Table 2 shows that the cervical angle in Group M became more kyphotic postoperatively (2 months, −4.6 ± 8.0°; 1 year, −4.7 ± 8.8°) than before surgery (−5.7 ± 9.1°). Although the cervical angle decreased slightly from 2 months to 1 year postoperatively, Group M became kyphotic at the last follow-up compared to before surgery. On the contrary, Group B showed continuous lordotic change after surgery (preop, −9.0 ± 10.1°; 2 months, −11.8 ± 11.5°; 1 year, −13.0 ± 12.3°) (Table 2). These losses of lordosis in Group M can be associated with cervical instability with excessive facetectomy.

Importantly, our results also present that BESS PCF can achieve foraminal enlargement as open PCF with a lower proportion of facet resection (Table 3). The facetectomy amount of open micrcoscopic PCF was nearly 50% (lengths, 51.6 ± 11.3%; area, 48.1 ± 10.4%). Zdeblick, T A et al. showed that extensive facet joint resection (>50%) may result in a 15–18% increase in lateral bending and rotation and thus instability [27]. An additional significant finding is that extensive facet joint resection induces larger segmental ROM in Group M than in Group B (Figure 6) (Table 2). The representative patient in Figure 6 reported cervical instability postoperatively. The facetectomy amount of open microscopic PCF is likely to cause larger segmental ROM and cervical instability. Conversely, BESS PCF involves relatively less facetectomy, resulting in a mean segmental ROM of 6.2 ± 5.6° in Group B.

We found that the extensive facet joint resection in open microscopic PCF was related to the surgical route. In open surgery, the extent of facet joint resection is proportional to the degree of foraminal decompression required due to the limited surgical corridor available [30]. This is the reason why the facetectomy amount of open microscopic PCF was more than BESS PCF. Figure 7 showed that a lateromedial surgical route was limited in open microscopic PCF. Endoscopic systems in PCF provide surgeons with direct visualization within the operating space, offering a significant advantage over open approaches. The small-diameter endoscopes used in this technique allow for improved viewing of the foraminal space through the undersurface of the resected facet joint, with fewer limitations compared to conventional methods. Leveraging the free movement of endoscopic views, surgeons can achieve adequate foraminal decompression by undercutting the facet joint while preserving the joint and its capsule, thus maintaining cervical stability.

Our study revealed that the operation time in Group B was longer than in Group M (Table 1). This finding contrasts with several studies comparing open microscopic PCF and BESS PCF at a single level, where the opposite trend was observed [5]. However, despite the shorter operation time, Group M exhibited a numerically longer mean hospital stay compared to Group B, although this difference did not reach statistical significance (Table 1, *p* = 0.12). This trend may be attributed to the greater extent of muscle dissection and tissue trauma involved in open microscopic PCF. The open technique typically requires extensive muscle stripping and retraction, as well as a longer incision, which may necessitate additional wound care and could potentially delay recovery. In contrast, BESS PCF employs a minimally invasive approach that preserves surrounding soft tissues, potentially facilitating quicker recovery and earlier discharge. While not statistically significant, these findings underscore potential trade-offs between procedural efficiency and postoperative recovery, which should be carefully weighed when selecting a surgical approach for multi-level cases. Endoscopic cervical surgery is challenging due to complex muscle and fascia layers. Each level requires new portals through dense tissue, risking obstruction even with working sheaths [31]. Consequently, the process of creating portals in multi-level BESS PCF is significantly more time-consuming, particularly due to the necessity of establishing new portals for each level. Absolute indications for two-, three-, or four-level surgeries have not been clearly defined, as surgical decisions should be tailored to individual factors such as pathology, anatomy, and surgical goals. For single-level procedures, BESS offers notable advantages by minimizing tissue trauma and enabling precise decompression, making open PCF less ideal in such cases. For two-level surgeries, advancements like the “sliding technique” in BESS, which we also utilize, further enhance its adaptability [32]. For three-level surgeries and beyond, open PCF often provides a practical advantage with its single-incision approach, facilitating efficient access to multiple levels. However, when preserving alignment and reducing tissue damage are priorities, BESS may still be preferable in select cases.

Although this study provides important insights, several limitations should be acknowledged. Firstly, its retrospective design with a single institution setting and single surgeon experience inherently restricts the ability to establish causal relationships and limits generalizability while introducing potential biases [33]. Secondly, our study has a relatively small number of patients included, which may constrain the generalizability of our findings. This limited sample size might affect the robustness of our results, emphasizing the need for future studies with larger cohorts to validate these findings and enhance their applicability in broader clinical settings. Furthermore, larger cohorts are essential for a more detailed analysis of the type and frequency of complications associated with different surgical approaches, particularly in multi-level posterior cervical procedures. While three-level surgeries are more common, including four-level cases in future studies could provide valuable insights, as these rare procedures inherently carry unique risks. These additional studies would help to better characterize complication profiles, compare outcomes across different surgical techniques, and refine the indications for each method, ultimately contributing to more comprehensive clinical guidance. Thirdly, we acknowledge that multi-level procedures can potentially increase the risk of iatrogenic instability. This risk was carefully considered during our surgical planning and execution. While our patient selection criteria excluded cases with pre-existing instability or those at high risk for postoperative kyphosis, the inherent nature of multi-level posterior cervical foraminotomy (PCF) carries a potential risk of iatrogenic instability. We made every effort to minimize this risk by aiming to preserve more than 50% of the facet joints and maintaining the joint capsule integrity during the procedure. However, this limitation should be noted as a factor that could influence the long-term outcomes of the procedure. Finally, we followed patients for a minimum of 1 year. Therefore, the long-term outcomes of open microscopic PCF and BESS PCF remain incompletely understood. Degenerative changes in the cervical spine and their effects on range of motion (ROM) and clinical outcomes can progress over extended periods. To fully elucidate the long-term implications of PCF on cervical spine biomechanics and patient quality of life, studies with longer follow-up durations are necessary [34,35].

## 5. Conclusions

In conclusion, while it is challenging to determine the superiority of one approach over the other, BESS-PCF may offer advantages in surgical route selection, enabling more tailored facet resection and targeted decompression. This could reduce the extent of facet joint resection and muscle stripping compared to open microscopic PCF, potentially contributing to better preservation of cervical stability and reduced postoperative neck pain. However, given the retrospective design and limited sample size of our study, further prospective research is needed to validate these observations and clarify the relative benefits of each technique.

## Figures and Tables

**Table 1:** Baseline characteristics.

	Group B (*n* = 30)	Group M (*n* = 30)	*p*
Age	57.4 ± 8.7	56.2 ± 6.7	0.74
Sex (M:F)	21:9	23:7	0.77
BMD	−0.6 ± 1.4	−0.5 ± 1.3	0.28
DM	8 (26.7%)	5 (16.7%)	0.53
Smoking	8 (26.7%)	8 (26.7%)	1.0
CAD	2 (6.7%)	4 (13.3%)	0.66
Number of levels			0.31
2	19 (63.3%)	16 (53.3%)	
3	11 (36.7%)	12 (40.0%)	
4	0 (0.0%)	2 (6.7%)	
Level			0.95
C4–5	9	12	
C5–6	24	26	
C6–7	24	24	
C7–T1	14	14	
Pathology			0.60
Soft disc	16 (53.3%)	13 (43.3%)	
Osteophyte	14 (46.7%)	17 (56.7%)	
24 h—drainage (mL)	53.4 ± 17.3	44.6 ± 14.7	0.13
Hospital stay	5.2 ± 1.7	6.3 ± 1.4	0.12
Op time	84.3 ± 11.9	61.7 ± 13.2	<0.01 *

BMD: bone mineral density; DM: diabetes mellitus; CAD: coronary artery disease. * Indicates *p* value < 0.05.

**Table 2:** Comparison of radiological outcomes between two groups.

	Group B (*n* = 30)	Group M (*n* = 30)	*p*
Pre			
Segmental Angle	2.8 ± 5.4	2.4 ± 4.6	0.38
Cervical Angle	−9.0 ± 10.1	−5.7 ± 9.1	0.19
C2–C7 SVA	21.6 ± 12.5	19.9 ± 9.5	0.81
ROM Segmental Angle	17.1 ± 8.4	18.2 ± 10.2	0.52
ROM Cervical Angle	42.7 ± 17.5	42.4 ± 14.9	0.93
2 M			
Segmental Angle	−4.3 ± 6.5	−0.5 ± 5.6	0.02 *
Cervical Angle	−11.8 ± 11.5	−4.6 ± 8.0	<0.01 *
C2–C7 SVA	17.9 ± 11.7	21.2 ± 9.5	0.21
ROM Segmental Angle	5.2 ± 5.5	9.2 ± 6.3	0.01 *
ROM Cervical Angle	18.0 ± 5.7	13.7 ± 6.4	0.21
1 Y			
Segmental Angle	−3.8 ± 6.1	−0.1 ± 4.6	0.01 *
Cervical Angle	−13.0 ± 12.3	−4.7 ± 8.8	<0.01 *
C2–C7 SVA	19.1 ± 10.6	19.8 ± 11.8	0.82
ROM Segmental Angle	6.2 ± 5.6	15.2 ± 7.5	<0.01 *
ROM Cervical Angle	28.5 ± 6.7	22.8 ± 6.0	0.64

* *p* < 0.05.

**Table 3:** Comparison of radiologic factors between two groups.

	Group B (*n* = 30)	Group M (*n* = 30)	*p*
Disc height (mm)	3.9 ± 0.8	3.6 ± 0.9	0.16
Gliding distance (mm)	1.4 ± 0.8	1.9 ± 3.3	0.45
Interspinous distance difference (mm)	4.2 ± 1.4	4.6 ± 1.6	0.23
Post-interspinous distance difference (mm)	4.4 ± 1.2	5.1 ± 1.9	0.07
Foraminal dimension (mm^2^)	34.3 ± 8.5	31.5 ± 7.8	0.16
Post foraminal dimension (mm^2^)	54.5 ± 8.2	53.3 ± 7.4	0.51
Foraminal enlargement (%)	63.8 ± 26.1	75.5 ± 34.6	0.13
Foraminal stenosis grade	3.6 ± 0.5	3.7 ± 0.4	0.10
Pfirmann grade	3.6 ± 0.5	3.8 ± 0.5	0.15
Bone bridge formation (*n*, %)	6 (20%)	16 (53.3%)	0.01 *
Facetectomy width (%)	43.2 ± 7.3	51.6 ± 11.3	0.01 *
Facetectomy area (%)	40.5 ± 8.1	48.1 ± 10.4	<0.01 *

* *p* < 0.05.

**Table 4:** Comparison of complications between two groups.

	Group B (*n* = 30)	Group M (*n* = 30)	*p*
Wound infection	0	1	
Hematoma	1	1	
Incomplete decompression	1	2	0.57
Local recurrence	1	0	
Postoperative seizure	0	0	
Delirium	0	0	
Total	3	4	0.68

## Data Availability

The raw data supporting the conclusions of this article will be made available by the authors upon request.
